# The Microbial Diversity and Biofilm Characteristics of d-PTFE Membranes Used for Socket Preservation: A Randomized Controlled Clinical Trial

**DOI:** 10.3390/jfb16020040

**Published:** 2025-01-23

**Authors:** Barbara Franović, Marija Čandrlić, Marko Blašković, Ira Renko, Katarina Komar Milas, Elitza Petkova Markova-Car, Bojana Mohar Vitezić, Dragana Gabrić, Ivana Gobin, Sabina Mahmutović Vranić, Željka Perić Kačarević, Olga Cvijanović Peloza

**Affiliations:** 1 Department of Anatomy, Faculty of Medicine, University of Rijeka, Braće Branchetta 20/1, 51000 Rijeka, Croatia; barbara.franovic@uniri.hr; 2 Doctoral School of Biomedicine and Health, Faculty of Medicine, University of Rijeka, Braće Branchetta 20/1, 51000 Rijeka, Croatia; 3 Department of Integrative Dental Medicine, Faculty of Dental Medicine and Health Osijek, J.J. Strossmayer University of Osijek, Crkvena 21, 31000 Osijek, Croatia; marija.candrlic@fdmz.hr; 4 Department of Oral Surgery, Faculty of Dental Medicine Rijeka, University of Rijeka, Krešmirova ulica 40/42, 51000 Rijeka, Croatia; marko_blaskovic@yahoo.com; 5 Dental Clinic Dr. Blašković, Linićeva ulica 16, 51000 Rijeka, Croatia; 6 Laboratory for Bioinformatics, Department of Biochemical Engineering, Faculty of Food Technology and Biotechnology, University of Zagreb, Pierottijeva ulica 6, 10000 Zagreb, Croatia; ira.renko@pbf.hr; 7 Center for Gut Microbiome, 10000 Zagreb, Croatia; 8 Department of Dental Medicine, Faculty of Dental Medicine and Health Osijek, J.J. Strossmayer University of Osijek, Crkvena 21, 31000 Osijek, Croatia; katarina.komar@gmail.com; 9 Interdisciplinary University Study of Molecular Biosciences, J.J. Strossmayer University of Osijek, Trg Sv. Trojstva 3, 31000 Osijek, Croatia; 10 Department of Basic and Clinical Pharmacology and Toxicology, Faculty of Medicine, University of Rijeka, Braće Branchetta 20/1, 51000 Rijeka, Croatia; elitza@medri.uniri.hr; 11 Department of Microbiology and Parasitology, Faculty of Medicine, University of Rijeka, Braće Branchetta 20/1, 51000 Rijeka, Croatia; bojana.mohar@uniri.hr (B.M.V.); ivana.gobin@uniri.hr (I.G.); 12 Department of Clinical Microbiology, Clinical Hospital Centre Rijeka, Krešimirova 42, 51000 Rijeka, Croatia; 13 Department of Oral Surgery, School of Dental Medicine, University of Zagreb, Gundulićeva 5, 10000 Zagreb, Croatia; dgabric@sfzg.hr; 14 Department of Dental Medicine, Clinical Hospital Centre Zagreb, 10000 Zagreb, Croatia; 15 Department of Microbiology, Faculty of Medicine, University of Sarajevo, 71000 Sarajevo, Bosnia and Herzegovina; sabina.mahmutovic@mf.unsa.ba; 16 Department of Anatomy, Histology, Embriology, Pathology Anatomy and Pathology Histology, Faculty of Dental Medicine and Health Osijek, J.J. Strossmayer University of Osijek, Crkvena21, 31000 Osijek, Croatia; 17 Botiss Biomaterials GmbH, 15806 Zossen, Germany

**Keywords:** membrane, randomized controlled trial, microbiology, qPCR, SEM

## Abstract

Background: Understanding microbial colonization on different membranes is critical for guided bone regeneration procedures such as socket preservation, as biofilm formation may affect healing and clinical outcomes. This randomized controlled clinical trial (RCT) investigates, for the first time, the microbiome of two different high-density polytetrafluoroethylene (d-PTFE) membranes that are used in socket preservation on a highly molecular level and in vivo. Methods: This RCT enrolled 39 participants, with a total of 48 extraction sites, requiring subsequent implant placement. Sites were assigned to two groups, each receiving socket grafting with a composite bone graft (50% autogenous bone, 50% bovine xenograft) and covered by either a permamem^®^ (group P) or a Cytoplast™ (group C). The membranes were removed after four weeks and analyzed using scanning electron microscopy (SEM) for bacterial adherence, qPCR for bacterial species quantification, and next-generation sequencing (NGS) for microbial diversity and composition assessment. Results: The four-week healing period was uneventful in both groups. The SEM analysis revealed multispecies biofilms on both membranes, with membranes from group C showing a denser extracellular matrix compared with membranes from group P. The qPCR analysis indicated a higher overall bacterial load on group C membranes. The NGS demonstrated significantly higher alpha diversity on group C membranes, while beta diversity indicated comparable microbiota compositions between the groups. Conclusion: This study highlights the distinct microbial profiles of two d-PTFE membranes during the four-week socket preservation period. Therefore, the membrane type and design do, indeed, influence the biofilm composition and microbial diversity. These findings may have implications for healing outcomes and the risk of infection in the dental implant bed and should therefore be further explored.

## 1. Introduction

Tooth extraction is one of the most common procedures in dental medicine. Following extraction, significant alveolar bone resorption occurs, reducing the dimensions of the alveolar ridge and creating unfavorable conditions for dental implant placement [1]. Studies have shown that the greatest amount of bone resorption occurs within the first month after extraction [2,3]. In the first six months after tooth extraction, the horizontal alveolar bone loss can range from 29% to 63%, while the vertical loss varies between 11% and 22%. Chappuis and colleagues [4] demonstrated an average bone loss of 3.8 mm in the buccolingual direction and a reduction of 1.24 mm in the initial height of the alveolar ridge. Additionally, the buccal wall of the alveolar bone resorbs rapidly, causing the ridge profile to shift lingually. This resorption is particularly pronounced when the buccal wall thickness is ≤1 mm, resulting in an even more substantial lingual shift in the ridge profile [4,5].

Socket preservation is widely recognized as an effective surgical approach to minimize bone resorption following tooth extraction, facilitate tissue regeneration, and prepare the site for future implant–prosthetic treatment. Studies have shown that the combination of a membrane with different bone grafts leads to greater preservation of the bone volume after tooth extraction compared with spontaneous healing alone [6,7,8,9,10,11]. The most common classification of barrier membranes is based on their chemical composition as either resorbable or non-resorbable [12]. Resorbable membranes offer the advantage of eliminating the need for a secondary surgical procedure, as they degrade spontaneously. However, as demonstrated by Rider and colleagues [13], resorbable membranes begin to lose strength and resistance within two weeks of implantation, with these properties being completely lost by the fourth week. In contrast, synthetic membranes are non-resorbable and require surgical removal during the fourth week of socket preservation. This requirement is a significant drawback, as the risk of infection at the implant site increases during membrane removal [14].

The first membrane used in socket preservation was expanded polytetrafluoroethylene (e-PTFE). However, e-PTFE membranes present certain drawbacks, including handling difficulties during fixation and their permeability to bacteria due to an open-pore microstructure [15]. In contrast, high-density polytetrafluoroethylene (d-PTFE) membranes feature a denser structure with a smaller pore size (0.2 µm), which prevents bacterial penetration and offers improved stability. In addition, due to their hydrophobicity, d-PTFE membranes have reduced microbial adhesion on their surface and do not need to be covered with soft tissue [16,17].

The oral microbiome consists of a diverse community of over 700 bacterial species, including commensal, symbiotic, and pathogenic types. These microorganisms form biofilms on both living and non-living surfaces. *Streptococci, actinomycetes*, and *veillonellae* are the primary colonizers of dental biofilms, creating a foundation for the attachment of other bacterial species. Changes in factors such as diet, oral hygiene, or the host immune response can alter the environment, promoting conditions that favor the growth of pathogenic species within the biofilm [18].

Currently, two of the most often used types of d-PTFE membranes in clinical practice differ in their physical properties. The permamem^®^ membrane (botiss biomaterials GmbH, Zossen, Germany) is a hydrophobic and soft d-PTFE membrane, whose smooth internal surface may reduce bacterial adhesion. In contrast, the Cytoplast™ membrane (Osteogenics Biomedical, Lubbock, TX, USA) features a rougher internal surface. These differences in surface texture and hydrophobicity play a critical role in bacterial adhesion and biofilm formation, as the surface characteristics significantly influence microbial interactions [19,20].

Understanding microbial interactions in vivo is important, as the composition of biofilms on membranes could affect healing outcomes, the risk of postoperative infections, and the long-term success of dental implants. Infection is a possible complication during alveolar ridge preservation procedures, as studies have reported early postoperative infections that could be attributed to pathogenic microbial colonization, with an incidence of 2.8% to 9.1% [21]. Biofilms that are dominated by pathogenic species, such as *Porphyromonas gingivalis* or *Prevotella intermedia*, have had negative impacts on the success of periodontal regeneration [22,23,24]. On the other hand, balanced microbial communities that are dominated by commensal species can promote supportive healing conditions [25]. In addition, it is known that specific pathogens such as the previously mentioned *P. ginigivalis* and *Fusobacterium nucleatum* are characteristic of dental implant loss [26].

To date, no randomized controlled clinical trials (RCTs) or in vivo studies have investigated the microbial diversity and biofilm characteristics of d-PTFE membranes, specifically permamem^®^ and Cytoplast™, following a 4-week open healing period for socket preservation. Considering the known differences in their microstructural properties and the variations in bacterial adherence that have been observed in vitro, we hypothesize that the type of membrane influences both the composition and structure of these oral biofilms. Therefore, this study aimed to evaluate these differences by examining the biofilm morphology using scanning electron microscopy (SEM), quantifying bacterial species with qPCR, and performing in-depth microbial profiling using next-generation sequencing (NGS).

## 2. Materials and Methods

### 2.1. Ethical Considerations

This RCT was conducted in accordance with the Declaration of Helsinki. The Ethics Committee of the Faculty of Medicine of the University of Rijeka (class: 003-05/20-1/151, no. 2170/29-02/1-20-2) approved the study of human participants who were willing to provide informed consent. This study was conducted in compliance with ethical standards and approved as an RCT, registered at ClinicalTrials.gov (NCT06694844). This study adhered to the CONSORT guidelines for reporting randomized controlled trials. The CONSORT Checklist has been provided as Supplementary Materials.

The confidentiality of the participants was rigorously maintained throughout this study. Prior to enrollment, each participant received comprehensive written and verbal information detailing this study’s objectives, methodology, and plans for result dissemination. Informed consent was obtained from all participants, who voluntarily agreed to participate after being fully briefed on this study’s procedures and requirements.

### 2.2. Sample Size Calculation

The minimum required sample size was calculated based on previous research [27,28,29,30], which indicated that each group should include at least 24 extraction sites to achieve a test power of 80% and a significance level of 0.05. This calculation was performed using the Power Analysis function in the Statistica™ program, version 14.0.1.25 (StatSoft, Tulsa, OK, USA, 2022), based on mean values and standard deviations. As some patients presented with multiple extraction sites, the sample size reflected the total number of sites rather than the number of individual patients to more accurately assess the outcomes.

### 2.3. Study Design, Patient Selection, and Materials

In order to address potential biases in patient selection, stringent measures were implemented. These measures are described in detail in the following section. Participant enrollment was conducted by M.B., who screened and identified eligible participants based on the inclusion and exclusion criteria. Patients requiring at least one tooth extraction and subsequent implant placement due to the failure of conventional therapies to preserve the affected teeth were included in this study. Before participation, all patients provided informed consent after reviewing the details of this study. Of the 56 patients who were initially screened, 39 met the inclusion criteria and were randomly allocated to one of two study groups. The random allocation sequence was generated by B.F. using a web-based randomization tool (https://www.randomizer.org/, accessed on 14 March 2022) [31]. The assignment of participants to the intervention groups was performed by M.B. based on this randomization process.

The participants included men and women over 18 years of age, who were all in good general health, with indications for tooth extraction due to vertical or horizontal fractures extending 2 mm or more apically from the gingival border, or due to failed endodontic or other conventional treatments. The exclusion criteria included patients with head and neck irradiation, those undergoing intravenous or oral bisphosphonate therapy, patients with untreated periodontal disease or uncontrolled diabetes, patients on immunosuppressive or long-term corticosteroid therapy, pregnant and lactating women, and patients who smoked more than 10 cigarettes per day [32,33].

All procedures were performed by a single experienced oral surgeon (M.B.). Prior to surgery, all participants received mechanical plaque and calculus removal and were instructed in oral hygiene practices. An oral antibiotic (Klavocin^®^ bid 1000 mg, Pliva, Zagreb, Croatia, or Clindamycin MIP^®^ 600 mg, Chem. Pharm. Fabrik GmbH, Ingbert, Germany, for penicillin-allergic patients) was administered one hour before surgery. Additionally, patients rinsed with a 0.2% chlorhexidine digluconate solution (Parodontax^®^ 0.2%, Brentford, London, UK) pre-operatively.

In both groups, a socket grafting procedure was performed immediately following tooth extraction using a composite bone graft comprising 50% autogenous bone and 50% bovine xenograft (cerabone*^®^*, botiss biomaterials GmbH, Zossen, Germany). After graft placement, the patients received one of two membranes, based on their pre-assigned group, as follows:

- Group P: in group P, the grafted site was covered with a d-PTFE membrane (permamem*^®^*, botiss biomaterials GmbH, Zossen, Germany).
- Group C: in group C, the site was covered with an alternative d-PTFE membrane (Cytoplast™, Osteogenics Biomedical, TX, USA).

After tooth extraction and curettage, the integrity of the buccal bone wall was assessed, and the patients were treated surgically based on the condition of the alveolus as follows:

(A) Preserved bony walls: If the alveolus had intact bony walls, the socket was filled with a 50:50 mixture of bovine bone biomaterial and autogenous bone, and then, a d-PTFE membrane was positioned beneath the buccal and lingual soft tissue flaps, covering 3*–*5 mm of the upper bone wall. The central portion of the membrane remained exposed, with no attempt to achieve primary closure.
(B) Partial resorption of the buccal wall: If partial vertical resorption of the buccal wall was observed, the membrane was positioned to cover at least 3 mm beyond the defect on the remaining buccal wall, extending apically, distally, and mesially. The socket was filled in the same way as in the above-described protocol, with a 50:50 mixture of bovine and autogenous bone. The membrane was stabilized along the lingual aspect of the alveolar bone, and, as in the “A” clinical situation, no primary wound closure was performed.

This approach allowed for tailored membrane coverage based on the degree of buccal wall preservation, optimizing the support for bone regeneration. Primary wound closure was intentionally avoided, as a buccal flap advancement for achieving primary soft tissue closure can lead to postoperative morbidity and alterations in the mucogingival line [34].

### 2.4. Surgical Protocol and Membrane Extraction

**Figure 1:**
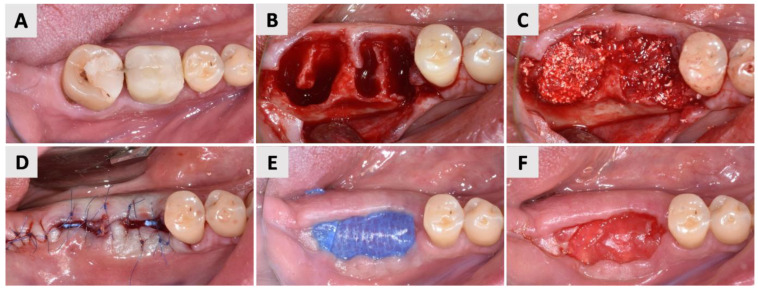
The clinical phases of the surgical procedure. (**A**) The initial clinical situation: an occlusal aspect of hopeless teeth 46 and 47 (FDI Notation System). The teeth were non-restorable due to untreatable periapical lesions. (**B**) The post-extraction sockets of teeth 46 and 47, showing a minimally elevated flap to reveal the buccal bone defect. (**C**) The sockets were grafted with a composite bone graft consisting of autogenous and xenogenic bone (cerabone*^®^*, botiss biomaterials, GmbH, Zossen, Germany). (**D**) As this patient was assigned to group P, a d-PTFE membrane (permamem*^®^*, botiss biomaterials, Zossen, Germany) was positioned to cover the extraction socket and buccal bone defect, with the flap being repositioned and secured with sutures, leaving a portion of the membrane intentionally exposed. (**E**) The healing site 4 weeks after socket preservation and prior to membrane removal. (**F**) Following membrane removal, a pseudoperiosteum covering the crestal portion of the extraction socket was left exposed, allowing healing to continue until implant placement at 6 months.

Following the pre-operative assessment, local anesthesia of 4% articaine hydrochloride with adrenaline at a concentration of 1:200,000 (Ubistesin*^®^* Forte, 3M, Neuss, Germany) was administered to two regions: the area indicated for tooth extraction (Figure 1A) and the retromolar region in the same jaw quadrant, which was selected for autogenous bone harvesting. After atraumatic extraction of the tooth, the socket was thoroughly debrided, and the integrity of the bone walls was assessed with a periodontal probe (15 UNC Color-Coded, Hu-Friedy, Chicago, IL, USA). Following an intrasulcular incision around the buccal surface of the adjacent teeth, a full-thickness mucoperiosteal flap was elevated (Figure 1B) using a surgical curette (Lucas 2.5 mm, Helmut Zepf, Seitingen-Oberflacht, Germany). After a thorough assessment of the buccal bone walls (see Section 2.3) and the required quantity of the autogenous bone, a full-thickness flap was elevated at the donor site. The autogenous bone was harvested using a Safescraper*^®^* Twist bone scraper (Geistlich Bone Harvesting Instruments, Basel, Switzerland). The donor site was subsequently closed with primary intention using standard suturing techniques (6.0 Surgicryl*^®^* Monofast, St. Vith, Belgium).

The extraction socket filling and membrane placement were carried out in accordance with the previously described protocol (Section 2.3). The grafting was carried out using the mixture of bovine xenograft (cerabone*^®^*, botiss biomaterials GmbH, Zossen, Germany) and autogenous bone (Figure 1C). Based on the group assignment, a d-PTFE membrane was used to cover the extraction socket—either permamem*^®^* (botiss biomaterials GmbH, Zossen, Germany) for group P or Cytoplast™ (Osteogenics Biomedical, TX, USA) for group C. The flap was repositioned and secured with non-resorbable 6.0 sutures (Surgicryl*^®^* Monofast, St. Vith, Belgium), leaving the central part of the membrane intentionally exposed (Figure 1D). Postoperatively, the patient was advised to continue the prescribed antibiotic therapy twice daily for 7 days, perform oral rinsing with 0.12% chlorhexidine solution twice a day, and use non-steroidal anti-inflammatory drugs as needed to manage pain.

Four weeks post-extraction, the d-PTFE membrane was removed in both groups, leaving the pseudoperiosteum covering the crestal part of the extraction socket exposed to support healing (Figure 1E*,*F). Healing was allowed to progress for six months, after which implant placement was performed in the prepared site. After extraction, the membranes were placed in a transport medium (Copan Group, Brescia, Italy) and immediately delivered to the Faculty of Medicine Rijeka for further analysis. Upon arrival, the membranes were rinsed with physiological solution and placed in an ultrasonic bath (BANDELIN electronic GmbH & Co. KG, Berlin, Germany) to remove dental biofilm. The obtained sonicate was then frozen at *−*80°C for storage (Heraaeus Group, Hanau, Germany) until further analysis.

The representative photographs of the clinical phase shown in Figure 1 are from group P. The same operative protocol as the one described in Figure 1 for group P was consistently applied to group C.

### 2.5. Scanning Electron Microscopy Analysis

To evaluate the bacterial adherence to the d-PTFE membranes, samples containing small pieces of the membrane that were cut off were prepared for SEM analysis immediately after the membrane removal. The membranes were rinsed in sterile phosphate-buffered saline and then air-dried in a high-flow sterile chamber (Ehret Bioban 190, Freiburg, Germany). The sample fixation was performed by immersing the membranes in a solution containing 4% glutaraldehyde and 0.5% paraformaldehyde (Sigma-Aldrich, Burlington, VT, USA) in 0.1 M phosphate-buffered saline (PBS, pH 7.2) (Sigma-Aldrich, Burlington, VT, USA) at 4 °C for 30 min. This fixation step preserves bacterial cells and membrane structure, ensuring stability during preparation for SEM analysis. After fixation, the samples underwent a graded dehydration process by immersion in increasing ethanol concentrations (50%, 70%, 80%, 90%, and 100%), with each immersion lasting 20 min. This step removes water from the samples to prevent morphological changes during SEM imaging under high-vacuum conditions. Each membrane was then mounted onto a sample holder using conductive carbon tape. To enhance the surface conductivity and prevent electron charging under the high-vacuum conditions of the electron beam, a thin 15 nm layer of gold palladium was applied using a precision etching and coating system (PECS II, Gatan Inc., Pleasanton, CA, USA). Finally, SEM imaging was conducted with a Jeol JSM-7800F field emission scanning electron microscope (JEOL Ltd., Tokyo, Japan), using an accelerating voltage of 7 kV and a working distance of 10 mm. No post-processing of the images was performed. The images are presented in the manuscript in their original, unmodified form.

### 2.6. Molecular Analyses: qPCR and Next-Generation Sequencing Methodology

#### 2.6.1. qPCR Analysis

##### Extraction of DNA Nucleic Acids

To avoid contamination during the preparation and extraction of the DNA, all actions were performed under sterile conditions. The personnel in charge of the preparation, isolation, and concentration measurements adhered strictly to protocols, using new sterile equipment for each step. Special attention was paid to protective gear, including gloves, lab coats, and masks [35]. To reduce the risk of mix-ups, the work was conducted in small batches, processing only two samples per round of extraction. The centrifugation steps were carried out in a large centrifuge (Eppendroff SE, Hamburg, Germany) with maximal separation space between samples. The tubes were kept closed and opened only when adding isolation buffers, and all pipetting steps were performed in a laminar flow cabinet (LANXESS AG, Cologne, Germany). Certified reagents were used, prepared as aliquots in advance, and a new DNA extraction kit was employed [36]. The extracted DNA was stored in sealed sterile tubes to avoid contamination, and the reopening of the tubes was minimized to reduce the chance of exposure.

DNA was isolated from the collected membrane sonicate samples using a Nucleospin Tissue Kit (Macherey Nagel, Duren, Germany) following a modified bacterial protocol. The sonicated samples were centrifuged at 15,000 rpm for 15 min in 1.5 mL Eppendorf tubes, after which the supernatant was removed, leaving approximately 50 μL of sediment. This sediment was resuspended in 250 μL of a prepared G+ lysis buffer, as specified by the manufacturer’s modified bacterial protocol, and vortexed thoroughly. Next, 40 μL of freshly prepared lysozyme solution was added, and the sample was incubated at 37 °C for 1 h. Following this, 30 μL of proteinase K was added, and the sample was incubated at 56 °C for 2 h. After incubation, 400 μL of B3 buffer was added, and the sample was vortexed and then incubated again at 70 °C for 15 min. The DNA was precipitated by adding 400 μL of chilled 98% ethanol. The manufacturer’s protocol was resumed for subsequent steps, with the final elution volume being reduced to 50 μL of BE. The concentration and purity of the extracted genomic DNA were assessed using a Qubit fluorometer (Thermo Fisher Scientific, Waltham, MA, USA).

##### qPCR Analysis

The quantification of bacteria in the membrane sonicate samples was performed on an Applied Biosystems 7500 Fast Real-Time PCR System (Applied Biosystems, Foster City, CA, USA), with specific TaqMan assays (Applied Biosystems, Foster City, CA, USA) targeting key bacterial species. These assays included bacteria of cariogenic significance, such as the Gram-positive facultative anaerobes *S. mutans* and *Streptococcus sobrinus*, as well as *Streptococcus salivarius*, which acts as an antagonist to these species. Additionally, the Gram-negative anaerobes *V. parvula* and *A. actinomycetemcomitans* were analyzed. A specific TaqMan assay for the 16s rRNA gene was used to represent the total bacterial load in the dental biofilm. TaqMan assay primers and probes were designed to specifically target the genes for each bacterium. For total bacterial quantification, the conserved region of the 16s rRNA gene was selected as described by Zibar Belašić et al. [37]. The qPCR reaction mixture (20 µL) consisted of 10 µL of TaqMan Master Mix, 1 µL of individual Custom TaqMan assays for each selected bacterium, 4 µL of water, and 5 µL of DNA sample diluted to 0.5 ng/µL. Each TaqMan assay contained specific custom-designed primers for amplifying the gtfB, gtfT, tnpA, lktA, and rpoB genes, as well as the 16s rRNA gene as a reference control [37]. Additionally, each assay included a custom-designed TaqMan fluorescently labeled probe with AM (fluorescein amidite) dye and a Q (quencher). The PCR conditions on the 7500 Fast Real-Time PCR System were as follows: initial incubation at 95 °C for 10 min for Taq DNA polymerase activation, followed by 50 cycles of 95 °C for 15 s (DNA denaturation) and 60 °C for 1 min (primer binding and extension). The reactions were carried out in 96-well plates with triplicates for each bacterium, alongside non-template controls for all genes.

The detection and quantification of the amplified genes for each bacterial species and the total 16s rRNA were based on changes in the fluorescence signal of the TaqMan probes. Fluorescence was emitted upon hybridization and hydrolysis of the probe at the target nucleic acid site, so an increase in fluorescence intensity indicated the level of gene amplification. The results were measured using a threshold cycle (Ct) value, which represents the required fluorescence level for detection and is consistently set within the exponential amplification phase. When sufficient DNA of the target gene is amplified, the fluorescent signal is detected, with the Ct value indicating the cycle in which the detection occurred. A higher Ct value corresponds to a lower initial concentration of the target gene in the sample, while a lower Ct value indicates a higher initial concentration.

qPCR was used to quantify the relative abundance of bacteria in the membranes. The ΔCt method was used to calculate the relative quantification of different bacterial populations, with the total bacterial load (16S rRNA equivalent) serving as the internal reference for each sample. ΔCt was calculated as the difference between the Ct value for a specific bacterial group and the Ct value for the total bacterial load (16S rRNA). Fold changes in bacterial abundance were expressed relative to the total bacterial load for each sample, representing the relative differences in bacterial populations across the two membrane types.

#### 2.6.2. Next-Generation Sequencing Analysis

For the purpose of amplicon sequencing of variable regions 3 and 4 of the 16S rRNA gene, DNA was isolated from the 20 membrane samples from group P and 16 membrane samples from group C and then sent to the Molecular Research Laboratory (MRDNA) in Texas, USA. Amplicon sequencing was performed using a set of primers 341F (5′-CCTAYGGGRBGCASCAG-3′) and 806R (5′-GGACTACNNGGGTATCTAAT-3′). The sequencing data were downloaded from “Illumina’s BaseSpace Sequence Hub” in the form of paired-end, demultiplexed fastq files. Data processing was performed using the software package Quantitative Insights Into Microbial Ecology 2 (QIIME2, v. 2024.2) [38]. The processing included filtering and denoising using the DADA2 program (integrated within QIIME2), concatenation of the sequences (since paired-end sequencing was used), and control for the presence of possible chimeras [39]. For the taxonomic determination of microorganisms, the Naive Bayes classifier tool in QIIME2 was applied, adapted to work with the SILVA database, version 138. The sequences were grouped according to the criterion of 99% similarity [40].

The diversity and richness of all samples were estimated based on alpha (Shannon index) and beta diversity (Bray–Curtis dissimilarity) using the seaborn, matplotlib, pandas, and matplotlib libraries and Python programming language, version 3.12, within the Pycharm environment [41,42]. Graphical representations of microbial species were also created using the Python programming language, version 3.12, within the Pycharm environment.

### 2.7. Statistical Analysis

The research data were analyzed using the conventional methods of descriptive statistics. The normality of the distribution was checked with the QQ plot. For all of the analyses, Python programming language, version 3.12, was used. To obtain probability, the seaborn, pandas, and matplotlib libraries within the Pycharm environment were used. A Mann–Whitney U test was used to compare selected bacteria. To determine the variability between samples, a principal component analysis was performed. Statistical differences in microbiota abundance between the two groups were considered if *p* < 0.05.

## 3. Results

**Figure 2:**
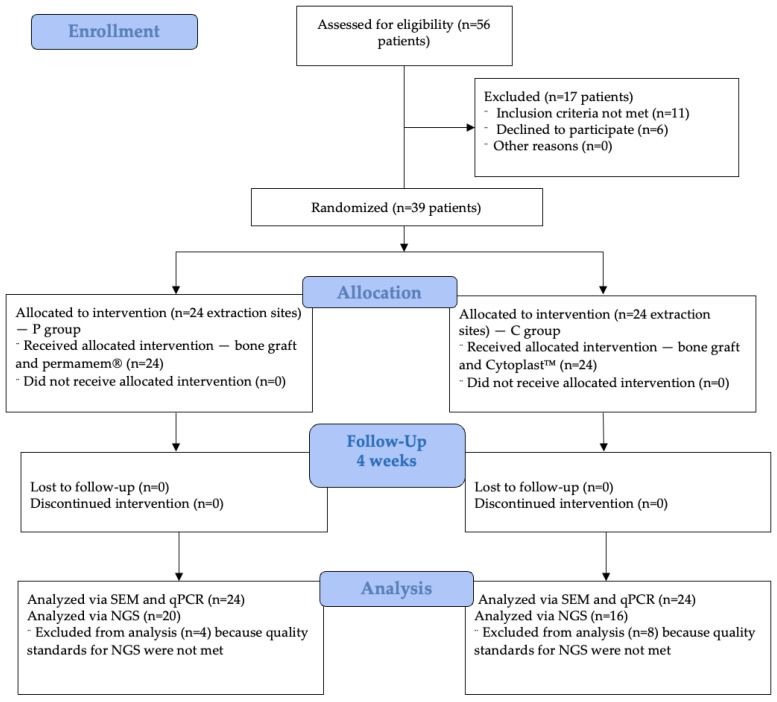
Flow diagram of this study.

A total of 56 patients were screened from the general population attending an oral surgery office between February and April of 2022. The surgical protocol was conducted between March and December of 2022. Group P consisted of 22 patients with 24 extraction sites, while group C included 17 patients, also with 24 extraction sites (Figure 2). The distribution of extraction sites is presented in Table 1.

The four-week healing period was uneventful, with only a few patients reporting minor complications such as pain and swelling. Finally, after this four-week period, the membranes were removed, and 24 samples from each membrane were processed for SEM and qPCR analysis. Of these, 20 samples from group P and 16 samples from group C met the quality standards for NGS. Samples that were not included in NGS analysis were excluded due to insufficient DNA yield, degraded quality, or contamination during preparation, ensuring that only high-quality samples contributed to the final analysis. The exclusion of low-quality samples was necessary to ensure that only high-quality data contributed to the final analysis. Although this reduced the sample size for the NGS analysis (to 20 samples in group P and 16 samples in group C), the sensitivity of the NGS method allowed for microbial diversity and composition assessments. Additionally, the complementary use of qPCR provided further validation of the results, avoiding any potential limitations caused by the exclusions.

### 3.1. Results of Scanning Electron Microscopy Analysis

**Figure 3:**
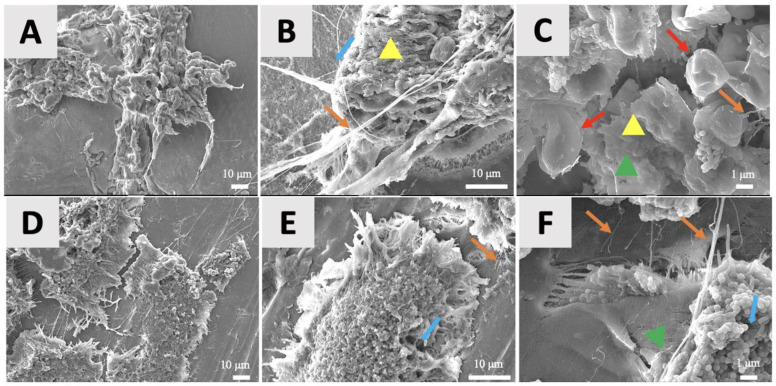
Representative scanning electron microscopy images of biofilms formed on membranes from group C (**A**–**C**) and P (**D**–**F**) at different magnifications: 800× (**A**,**D**), 2000× (**B**,**E**), and 5000× (**C**,**F**). (**A**–**C**) The representative C membrane shows a dense extracellular matrix network (orange arrows), with strands of extracellular material that is interwoven with tissue cells (red arrows). Clusters of cocci are scattered across the surface, often adhering to tissue cells and membrane regions. Polymeric encasements or “sweaters” (yellow triangles), which envelop bacterial cells, are also observed. (**D**–**F**) The representative P membrane displays mature mixed biofilm clusters with visible extracellular polymeric substance (green triangle) layers and strands of extracellular material. The blue arrows indicate water channels with varying openings, facilitating nutrient and waste exchange within the biofilm. The P membrane exhibits fewer tissue cells compared with the C membrane, although biofilms are observed on both the intact surfaces and damaged regions of the membrane.

The SEM microphotographs provided detailed visualizations of the surface topography and biofilm characteristics of the P and C membranes. SEM imaging revealed multispecies biofilms that were characterized by a combination of bacterial morphologies—rod-shaped, spiral-shaped, and cocci clusters—distributed across the membrane surfaces (Figure 3). Bacteria in biofilms can exhibit various morphological shapes that contribute to their structural organization and function. Cocci are spherical-shaped bacteria that often cluster together, forming dense microcolonies. Rod-shaped bacteria are elongated and cylindrical, commonly aligning to form filamentous structures within biofilms. Spiral-shaped bacteria have a helical or twisted form, which aids their motility and allows them to navigate biofilm environments. These distinct bacterial morphologies play a role in maintaining biofilm integrity and contributing to its complexity [43,44]. Unique topographical differences were noted between the two membranes. In group C (Cytoplast™) membranes, a dense extracellular matrix network was evident, with strands of extracellular material being interwoven among the tissue cells. Small cocci clusters adhered to both the tissue cells and membrane surface, with bacterial cells being encapsulated by polymeric layers, or “sweaters”, distributed across the matrix (Figure 3A–C). Group P (permamem*^®^*) membranes (Figure 3D,E), in contrast, exhibited mature biofilm clusters featuring smooth extracellular polymeric substance layers and distinct water channels with varying diameters. Biofilms were observed on both intact membrane surfaces and areas with structural imperfections. However, the presence of tissue cells on group P membranes was notably lower than on group C membranes.

### 3.2. Microbial Diversity and Taxonomic Analysis

#### 3.2.1. Alpha and Beta Diversity (Next-Generation Sequencing)

**Figure 4:**
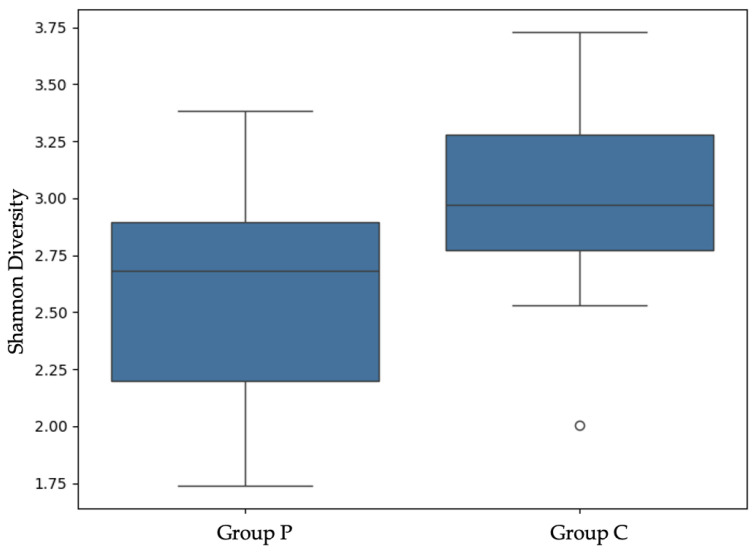
Alpha diversity. The results of the alpha diversity analysis indicate that group C exhibits significantly higher microbial diversity than group P (*p* = 0.019). In group C, the white dot is sample 9, which gives a much lower Shannon Diversity index value than the rest (1.933). This may indicate a less diverse microbial community compared to other samples in this group. Alpha diversity, measured using the Shannon index, reflects both the variety of microbial species (richness) and the balance in their relative abundance (evenness) within a sample. The higher Shannon index in group C suggests a greater variety of microbial species and a more even distribution of the microbial community compared with group P.

**Figure 5:**
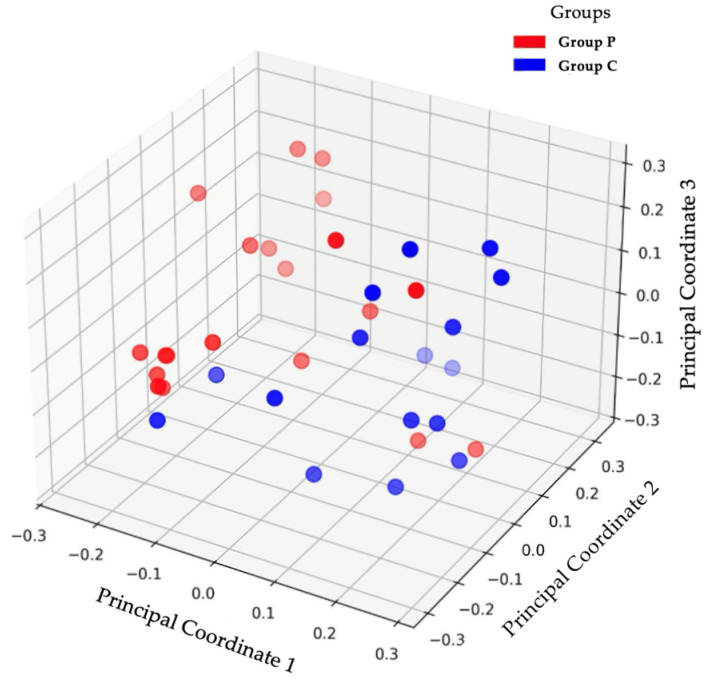
The results of the beta diversity analysis indicate no statistically significant difference between the microbiota of the two groups (*p* = 0.512). Beta diversity measures differences in microbial community composition between samples, providing insights into how distinct or similar the microbiota are across groups. Although the *p*-value suggests no statistically significant variation, the spatial separation of group P and C samples in the analysis suggests a difference in the overall composition of the microbiota of these groups. Shading is used to improve the presentation of three-dimensionality and to clarify the spatial positioning within the figure.

In order to compare the microbial diversity within and between samples, the alpha and beta diversities of the group P and C samples were calculated. The calculations and graphical representation were made at the taxonomic level of species in order to determine any potential statistical significance (Figure 4 and Figure 5).

#### 3.2.2. Taxonomic Analyses (Next-Generation Sequencing and qPCR)

**Figure 6:**
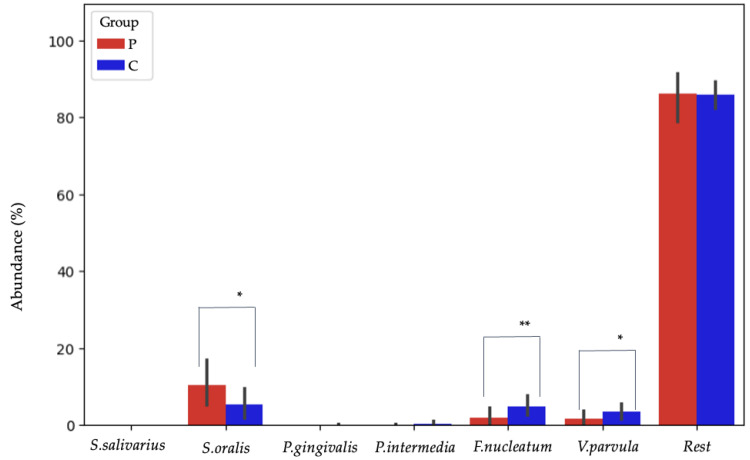
Bacterial abundance at the species level in groups P and C, detected by means of next-generation sequencing. Among the analyzed bacteria, *S. oralis* (*) was the most abundant species in both groups, with a higher abundance in group P. *F. nucleatum* (**) and *V. parvula* (*) showed higher abundances in group C compared with group P. In this context, “abundance” refers to the proportion of a specific bacterial species within the total microbial community of a group. The abundances of *S. salivarius* (group P: 0.019%; group C: 0.045%), *P. gingivalis* (group P: 0.001%; group C: 0.099%), and *P. intermedia* (group P: 0.073%; group C: 0.388%) are lower than 20% and are therefore not visible in the figure. * *p* < 0.05; ** *p* < 0.01.

Using 16S rRNA sequencing, *S. salivarius*, *S. oralis*, *P. gingivalis*, *P. intermedia*, *F. nucleatum*, and *V. parvula* were detected from the selected species. Other bacterial species were classified in the “Rest” group (Figure 6).

The differences between group P and C samples were monitored at the species level for the bacteria *S. mutans*, *S. salivarius*, *S. oralis*, *S. sobrinus*, *A. actinomycetemcomitans*, *P. gingivalis*, *P. intermedia*, *F. nucleatum*, and *V. parvula*. *S. oralis* emerged as the most prevalent species in both groups, with a significantly higher abundance observed in group P compared with group C (Figure 6). In group C, the least present species was *S. salivarius*, while in group P, it was *P. gingivalis*. Statistical significance was not confirmed by comparing the abundances of these bacteria between the groups. Among other bacteria, *F. nucleatum* and *V. parvula* were significantly more represented in group C (Table 2, Figure 6).

The detection of *S. mutans*, *S. salivarius*, *V. parvula*, *S. sobrinus*, and *A. actinomycetemcomitans* was achieved using the qPCR method. A total of 24 group P and 24 group C samples were analyzed.

**Figure 7:**
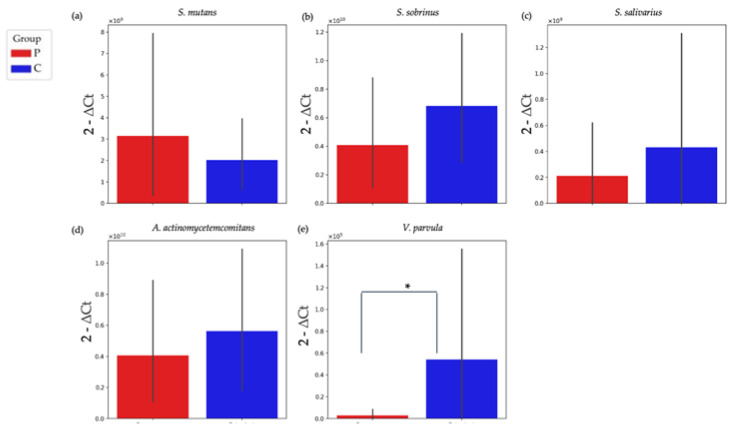
qPCR analysis of selected bacterial species. The figure presents the distribution of bacterial species, quantified by means of qPCR in groups P (red columns) and C (blue columns). The analyzed species include (**a**) *S. mutans*, (**b**) *S. sobrinus*, (**c**) *S. salivarius*, (**d**) *A. actinomycetemcomitans*, and (**e**) *V. parvula*. Among all tested bacteria, the largest difference between the groups was observed for *V. parvula*, which showed a higher abundance in group C than in group P (*p* = 0.044, graph “e”). * *p* < 0.05.

At the level of all tested bacteria, the largest difference was observed in the case of *V. parvula* bacteria, while the smallest differences between the groups were observed in *S. mutans*. Similarly to the results of the NGS, *V. parvula* showed an increased abundance in group C compared with group P (Figure 7).

## 4. Discussion

In this RCT, we investigated the microbial biofilm composition and diversity of two different d-PTFE membranes, P (permamem^®^) and C (Cytoplast™), four weeks post socket preservation. Through SEM, qPCR, and NGS analyses of the bacterial adherence, species quantification, and microbial diversity, we aimed to determine whether the choice of membrane influences microbial dynamics.

The presented RCT features a unique design and is the first study to explore microbial diversity at a high molecular level in clinical settings of open healing with d-PTFE membranes for tissue regeneration. The open healing approach offers multiple benefits, including the preservation of the mucogingival border, the promotion of the formation of a wider band of keratinized mucosa after healing, and the minimization of postoperative discomfort, with the patients reporting reduced swelling and pain [45,46]. A similar study design was recently presented by Mazzucchi et al. [47], who analyzed d-PTFE membranes using SEM and the BioTimer™ assay four weeks after socket preservation, but their analysis did not extend to an in-depth investigation of the microbial genome, as performed in our study. Mazzucchi et al. reported that d-PTFE membranes were colonized on both sides by microbes, but the overall bacterial load was low, allowing the healing process to proceed uneventfully and without infection, which aligns with our findings of a complication-free healing period.

One of the key advantages of non-resorbable membranes is their availability for use in open healing approaches, which offer several benefits in regenerative procedures involving the alveolar bone and mucosa [48,49]. However, membrane exposure in these approaches can serve as a reservoir for microorganisms, increasing the risk of infection and potentially triggering the recurrence of periodontitis, particularly in patients with elevated levels of periodontal pathogens [50,51]. A recent study by Gil et al. [52] introduced a novel 3D-printed apparatus to evaluate the bacterial adherence and penetration of d-PTFE and collagen membranes. Their findings confirmed that bacterial adherence and penetration vary significantly among different membrane types, demonstrating that d-PTFE membranes showed resistance to bacterial penetration while maintaining their surface adherence, which is essential because they serve as physical barriers during tissue regeneration [53].

Biofilm architecture refers to the structural organization of the microorganisms that form a biofilm [54]. Biofilms are a nonuniform, arranged group of microorganisms living within an extracellular polymeric substance (EPS) matrix that the group itself produces [55]. They adhere to each other and exist on living or non-living surfaces. Biofilms exist almost everywhere, inhabiting medical implants, living tissues, water channels, pipes, hospital floors, food processing units, and other biotic and abiotic surfaces [56,57]. The architecture of a biofilm has a significant impact on its biological functions, ecological roles, and resistance to external factors. It is important for protection, resource utilization, and microbe–microbe interactions. Therefore, structural knowledge regarding biofilms is of great importance in order to understand the survival and behavioral strategies of biofilms [58,59,60,61]. Keeping this in mind, the SEM results revealed diversity in the biofilm compositions and surface characteristics of the two d-PTFE membranes. Multispecies biofilms, comprising rod-shaped and spiral-shaped bacteria and clusters of cocci, were observed on both group P and C membranes (Figure 3), reflecting the complex microbial ecosystems that are typical of the oral cavity [62,63]. Group C membranes demonstrated dense extracellular matrix networks and polymeric encasements, suggesting robust biofilm development with strong bacterial adhesion, particularly of cocci, on both the tissue cells and membrane surface. In contrast, group P membranes exhibited mature biofilm clusters, characterized based on their smooth EPS layers and water channels, but with fewer tissue cells adhering to the membrane. The observed structural differences in biofilm formation on the membranes align with findings from previous studies examining bacterial adhesion to d-PTFE membranes. In vitro research has demonstrated that variations in membranes’ microstructures, such as their crystallinity and surface roughness, significantly influence the bacterial adherence and biofilm development [19]. These findings suggest that the microstructural properties of the d-PTFE membranes play an important role in determining the biofilm development, which is consistent with the differences observed between group P and C membranes in the current study. It is well known that dense biofilms with high cell densities can create a protective microenvironment that enhances their resistance to external threats, such as antimicrobials and immune responses [64,65]. In contrast, the water channels in group P membranes might promote nutrient flow and waste removal, potentially avoiding microbial colonization [66].

The qPCR analysis revealed the bacterial dynamics on the d-PTFE membranes during the four-week healing period, emphasizing differences in the microbial composition between the group P and C membranes. The qPCR analysis demonstrated no statistically significant differences in the relative abundance between groups P and C for most of the tested bacterial species (*S. mutans*, *S. salivarius*, *S. sobrinus*, and *A. actinomycetemcomitans*), with *p*-values exceeding 0.05. This indicates that the abundances of these bacteria were comparable across the two groups. However, a significant difference was observed for *V. parvula*, which exhibited a higher abundance in group C compared with group P (*p* = 0.044). *V. parvula* is a Gram-negative, anaerobic coccus that is commonly found in the human oral cavity. It has an important role in dental biofilm formation by metabolizing lactate produced by other oral bacteria, such as *Streptococcus* sp., into weaker acids, thereby influencing the overall pH and microbial composition of the biofilm [67]. This metabolic cross-feeding promotes a diverse microbial community and contributes to the maturation and stability of dental biofilms [68]. In the context of the dental biofilms on group P and C membranes, the higher abundance of *V. parvula* observed in group C suggests an environment that is supportive of biofilm maturation and metabolic cross-feeding, potentially enhancing microbial stability and resilience. This aligns with the dense extracellular matrix that was observed in the SEM analysis, which may further facilitate the role of *V. parvula* as a bridge species in the development of complex biofilm architectures.

The presence of *S. mutans* and *A. actinomycetemcomitans*, key contributors to carcinogenicity and periodontal disease [69,70], exhibited only minor differences between the groups, indicating that their colonization was not significantly influenced by the membrane type under the studied conditions. *S. salivarius* is a species that is recognized for its antagonistic effects against pathogenic bacteria [71]. Building upon our in vitro findings [72] on the modulatory role of *S. salivarius* in mixed biofilm environments, the present in vivo study further highlights the influence of bacterial interactions on biofilm composition and diversity in vivo. In this context, its comparable presence across both group P and C membranes reflects that its role in maintaining oral biofilm homeostasis is consistent, regardless of the membrane type.

Alpha and beta diversity are fundamental principles in microbial research and are used to define and describe the diversity within a sample and between samples. Alpha diversity provides insights into the differences within a sample, considering the structure of the microbial community in terms of the number of taxonomic groups, their distribution, or both. Beta diversity provides insights into the differences between samples, considering the presence/absence of certain sequences or their relative abundance. Both levels can be represented using various metrics, such as the Shannon index for alpha diversity and Bray–Curtis dissimilarity for beta diversity [73,74,75].

Alpha diversity revealed that group C membranes supported higher microbial diversity and a more even distribution of microbial species. A balanced microbial biofilm, characterized by diverse species, is often associated with health by fostering competitive microbial interactions and limiting the dominance of pathogenic species. This balance contributes to functional stability and resilience within a biofilm community [76,77]. In the context of the oral cavity, the greater diversity observed on group C membranes may foster an environment that is less conducive to pathogenic dominance, potentially supporting healthier healing outcomes. However, the presence of *F. nucleatum* in the group C samples raises important considerations, as this bacterium is a key pathogen associated with biofilm maturation and periodontal disease [78,79]. While diversity generally implies a balanced ecosystem, the inclusion of opportunistic pathogens can disrupt the balance between the biofilm and the host, increasing the risk of infection [80]. In addition, these findings have possible clinical implications. Higher microbial diversity, as seen on group C membranes, can promote biofilm stability and resilience, fostering competitive microbial interactions that may limit the overgrowth of specific pathogenic species, and thereby supporting healing. However, the presence of opportunistic pathogens such as *F. nucleatum* in the group C samples raises concerns, as such bacteria are linked to periodontal disease, biofilm maturation, and even dental implant loss [26,78,79,81]. The dense extracellular matrix observed on group C membranes may further enhance bacterial adhesion and resistance, potentially influencing infection risks [21]. This emphasizes the role of biofilm diversity in clinical outcomes, and therefore, future research should integrate long-term clinical outcomes, such as bone volume changes, histological analyses, and implant stability, to complement microbial findings.

The beta diversity analysis of the NGS results revealed distinct patterns in the structural grouping and microbiota uniformity between the group P and C membranes (Figure 5). The group P samples (red) displayed partial grouping but with notable scattering, reflecting a moderate level of homogeneity within the group, coupled with substantial inter-sample variation. Conversely, group C samples (blue) exhibited tighter grouping with minimal scattering, indicating greater uniformity in the microbial composition across samples within this group.

A comparison between the NGS and PCR results shows differences in the detection of specific bacterial species. Certain bacteria that were identified through PCR, such as *A. actinomycetemcomitans*, were not detected using the 16S rRNA sequencing method used in NGS. However, an analysis at higher taxonomic levels of the NGS results revealed the presence of the *Aggregatibacter* genus, suggesting that while species-level identification was not achieved, the genus was still represented in the samples. Several factors could explain these discrepancies. PCR is highly sensitive and capable of detecting specific genes even at low concentrations, making it effective for identifying bacteria that are present in minimal quantities [82,83]. On the other hand, NGS, which sequences the entire sample, may not have a sufficient number of specific sequences to capture them [84]. Furthermore, PCR uses specific primers for amplification, while NGS sequences all the present ones. For example, if some bacteria are present but contain genetic variants that are not detected by means of NGS, this could be the reason for the differences in results. Moreover, if the detection threshold is set too high, bacteria that are present in smaller amounts may not be identified by means of NGS. In addition, if the sample for NGS is contaminated or insufficiently prepared, this can affect the success of sequencing and the detection of bacteria that can be detected by means of PCR [85,86,87]. Therefore, our findings reveal the complementary nature of PCR and NGS in oral microbial analysis, as both of these methodologies have strengths and limitations in identifying bacterial species within complex biofilms.

It is necessary to address why we decided against using a non-intervention control group, whose sockets would heal spontaneously, in this study. This is because, as previous research studies have demonstrated, an absence of socket preservation leads to significantly greater volume loss compared with the application of preservation techniques [88,89]. In addition, the inclusion of a non-intervention group was not possible due to the primary focus of this study, which was to analyze microbial biofilms on d-PTFE membranes. This is a process that cannot occur in sockets that are left to heal naturally. Furthermore, ethical and clinical considerations played a significant role, as it is known that alveolar ridge preservation procedures significantly reduce bone resorption compared with spontaneous healing [90,91,92]. Providing patients with the best available treatment aligns with ethical and clinical standards that prioritize their well-being.

## 5. Conclusions

This study has certain limitations that should be acknowledged. Specifically, it does not include long-term follow-up data on clinical outcomes, as we have mentioned previously. This includes radiographic bone volume changes, histomorphometric analyses, implant stability, and long-term implant success. Future research should incorporate these parameters to better understand the relationship between the biofilm characteristics of d-PTFE membranes and their impact on bone regeneration and overall treatment success in clinical settings. Performing swabs before surgery and after membrane removal would help to determine whether the colonizing species originated from the oral environment or emerged post-operatively. However, our study focused specifically on membrane-associated bacterial adherence. Future studies incorporating pre- and post-surgical swabs could provide insight into the influence of baseline oral microbiota on membrane colonization.

Despite these limitations, the findings of this study shed light on the biofilm characteristics of two types of d-PTFE membranes in vivo for the first time. The results demonstrate that the membrane type does, indeed, influence the biofilm composition and microbial diversity, with group C membranes showing higher microbial diversity but also the presence of opportunistic pathogens such as *F. nucleatum*. These findings emphasize the importance of optimizing membrane designs to promote favorable microbiome interactions while minimizing infection risks. Practitioners should consider the balance between the microbial diversity and pathogenic load when selecting non-resorbable membranes for guided bone regeneration, ultimately aiming to support the optimal clinical settings for tissue healing.

## Figures and Tables

**Table 1:** Extraction site distribution.

Extraction Site	Group P (n = 24)	Group C (n = 24)
Mandibular molars and premolars	11	7
Maxillary molars and premolars	12	12
Maxillary incisors	1	5

**Table 2:** Bacterial species detected by means of next-generation sequencing and qPCR.

Next-Generation Sequencing		qPCR	
Bacterium	*p*-Value	Bacterium	*p*-Value
*S. oralis*	0.043	*S. mutans*	0.870
*S. salivarius*	0.309	*S. salivarius*	0.274
*V. parvula*	0.023	*V. parvula*	0.044
*P. gingivalis*	0.084	*S. sobrinus*	0.170
*P. intermedia*	0.475	*A. actinomycetemcomitans*	0.521
*F. nucleatum*	0.010		

*p* < 0.05 implies statistical significance.

## Data Availability

The original contributions presented in this study are included in the article and Supplementary Materials; further inquiries can be directed to the corresponding authors.

## Supplementary Materials

The following supporting information can be downloaded at https://www.mdpi.com/article/10.3390/jfb16020040/s1.
